# Spontaneous generation of prions and transmissible PrP amyloid in a humanised transgenic mouse model of A117V GSS

**DOI:** 10.1371/journal.pbio.3000725

**Published:** 2020-06-09

**Authors:** Emmanuel A. Asante, Jacqueline M. Linehan, Andrew Tomlinson, Tatiana Jakubcova, Shyma Hamdan, Andrew Grimshaw, Michelle Smidak, Asif Jeelani, Akin Nihat, Simon Mead, Sebastian Brandner, Jonathan D. F. Wadsworth, John Collinge

**Affiliations:** 1 MRC Prion Unit at UCL, UCL Institute of Prion Diseases, London, United Kingdom; 2 Department of Neurodegenerative Disease, UCL Queen Square Institute of Neurology and Division of Neuropathology, the National Hospital For Neurology and Neurosurgery, University College London NHS Foundation Trust, Queen Square, London United Kingdom; Scuola Internazionale Superiore di Studi Avanzati, ITALY

## Abstract

Inherited prion diseases are caused by autosomal dominant coding mutations in the human prion protein (PrP) gene (*PRNP*) and account for about 15% of human prion disease cases worldwide. The proposed mechanism is that the mutation predisposes to conformational change in the expressed protein, leading to the generation of disease-related multichain PrP assemblies that propagate by seeded protein misfolding. Despite considerable experimental support for this hypothesis, to-date spontaneous formation of disease-relevant, transmissible PrP assemblies in transgenic models expressing only mutant human PrP has not been demonstrated. Here, we report findings from transgenic mice that express human PrP 117V on a mouse PrP null background (117VV Tg30 mice), which model the *PRNP* A117V mutation causing inherited prion disease (IPD) including Gerstmann-Sträussler-Scheinker (GSS) disease phenotypes in humans. By studying brain samples from uninoculated groups of mice, we discovered that some mice (≥475 days old) spontaneously generated abnormal PrP assemblies, which after inoculation into further groups of 117VV Tg30 mice, produced a molecular and neuropathological phenotype congruent with that seen after transmission of brain isolates from IPD A117V patients to the same mice. To the best of our knowledge, the 117VV Tg30 mouse line is the first transgenic model expressing only mutant human PrP to show spontaneous generation of transmissible PrP assemblies that directly mirror those generated in an inherited prion disease in humans.

## Abstract 2

Transgenic mice expressing the human prion protein containing a mutation linked to the inherited prion disease Gerstmann-Sträussler-Scheinker disease develop spontaneous neuropathology. This represents the first human prion protein transgenic model to show spontaneous generation of transmissible prion assemblies that directly mirror those generated in humans.

## Introduction

Prions are lethal infectious agents that cause fatal neurodegenerative diseases in mammals, including Creutzfeldt-Jakob disease (CJD) in humans, scrapie in sheep and goats, and bovine spongiform encephalopathy (BSE) in cattle [1–3]. They are unique pathogens and are composed of infectious assemblies of misfolded host-encoded prion protein (PrP), some of which acquire protease-resistance and are classically designated as PrP^Sc^ [1,2]. Prions propagate by means of seeded protein polymerization, a process that involves the addition of PrP monomers to an elongating assembly of misfolded PrP chains followed by fission of the polymer to produce more seeds. Different prion strains producing distinct disease phenotypes can propagate in the same inbred host and appear to be encoded by distinct PrP conformations and assembly states [1,2,4–9].

Human prion diseases are associated with a range of clinical presentations, and they are classified by both clinicopathological syndrome and aetiology, with subclassification according to molecular criteria [3,6,10–14]. Approximately 15% of cases are associated with autosomal dominant pathogenic mutations in the human prion protein gene (*PRNP*), and, to date, more than 40 mutations have been described [13,15–17]. These include insertions of between 4 and 12 extra repeats within the octapeptide repeat region between codons 51 and 91, a 2-octapeptide repeat deletion and various other mutations causing missense or stop substitutions or other insertions with and without a frameshift. How pathogenic mutations in *PRNP* cause inherited prion disease (IPD) has yet to be resolved; however, in most cases, the mutation is thought to lead to spontaneous conformational change in the expressed protein, leading to the generation of disease-related PrP assemblies that propagate by seeded protein misfolding. Although a wealth of data from acquired or sporadic CJD indicates that residue 129 polymorphism of human PrP critically dictates thermodynamic preferences for PrP assemblies associated with distinct human prion strains [2,9,11,15,18–20], the full spectrum of effects that different pathogenic *PRNP* mutations have remains unclear. Notably a common feature of *PRNP* point mutations associated with conspicuous amyloid PrP plaque deposition in brain is that the expressed full-length mutant PrP forms 2 distinct disease-related assemblies of misfolded PrP. One assembly forms N-terminally truncated protease-resistant fragments that correspond to those generated from classical PrP^Sc^ (PrP 27–30 [1]), which is enriched in brain areas showing synaptic PrP deposition, spongiform vacuolation, and neurodegeneration. The other disease-associated assembly forms smaller N- and C-terminally truncated protease-resistant fragments (typically 7–15 kDa, derived from the central region of PrP), which is associated with PrP amyloid plaques [21–32]. Such distinct disease-associated PrP assembly states from Gerstmann-Sträussler-Scheinker (GSS) patients with the P102L PrP mutation transmit different phenotypes to experimental reporter mice resulting in either a lethal transmissible spongiform encephalopathy (associated with transmission of classical PrP^Sc^) or a clinically silent PrP amyloidosis (associated with the transmission of the PrP conformer generating an approximately 8 kDa, protease-resistant PrP fragment) [33–35]. Recently we proposed that authentic prions responsible for lethal transmissible spongiform encephalopathies are comprised of infectious paired fibre PrP rods (20 nm in width, generating proteolytic fragments corresponding to PrP 27–30) [36] and that in IPD patient brain distinct single PrP fibres (approximately 10 nm in width that generate N- and C-terminally truncated protease-resistant fragments) may co-propagate and account for the abundant amyloid PrP plaques that distinguish the IPD neuropathological phenotype [37,38]. Temporal and spatial differences in the propagation of paired fibre PrP rods or single amyloid PrP fibres within the brain could readily account for the diversity of clinicopathological phenotypes seen in family members with the same *PRNP* mutation [39–42].

Potential co-propagation of infectious, pathogenic, paired fibre PrP rods and distinct single amyloid PrP fibres in IPD patient brain places considerable constraints on the ability to accurately model these diseases in laboratory mice. Variation in the substructure of infectious PrP rods or the single amyloid PrP fibres in different IPDs (governed by the specific PrP missense mutation) may dictate their strain-specific biological properties and host range via conformational selection [2,9,18,43]. In particular, the intrinsic ability of a particular mutant PrP assembly (paired fibre PrP rod or single amyloid PrP fibre) to propagate at all may critically rely upon expression of the homotypic mutant PrP sequence. To date, however, much of the modelling of pathogenic mutations seen in IPD has involved the superimposition of the human PrP mutation onto rodent PrP [44–54] or bovine PrP sequences [55,56]. However, biophysical studies of an experimental mutation introduced into the mouse or human PrP sequence have shown profoundly dissimilar structural consequences for the expressed protein [57,58]. Indeed, recent modelling of the IPD *PRNP* P102L mutation using mice expressing the authentic mutant human PrP sequence strongly suggests that the presence of this mutation within the mouse PrP sequence leads to the generation of a novel experimental prion strain following challenge with human prions that is markedly different from the prion strain propagated in human disease [43].

Currently there has been no report of spontaneous neurological dysfunction in mice expressing IPD mutations directly in human PrP. In this regard, transgenic mice expressing human 102L PrP at 1.5- to 3-fold, human 200K PrP at 2- to 3-fold [59] or human 224V PrP at 1.5- to 3.2-fold [60] do not develop spontaneous disease or evidence for spontaneous generation of prions as they age. Recently, we demonstrated that IPD A117V (associated with GSS phenotypes in humans characterised by abundant PrP plaque deposition in brain [16,30,61]) is an authentic, transmissible, prion disease by demonstrating that inoculation of A117V patient brain to transgenic mice expressing mutant human PrP 117V (117VV Tg30 and Tg31 mice) not only results in transmission of abundant PrP amyloid plaque pathology in brain (associated with a characteristic disease-related PrP assembly generating an approximately 8 kDa protease-resistant PrP fragment analogous to that detected in A117V patient brain [27,31,32]) but also results in propagation of classical PrP^Sc^ assemblies (generating proteolytic fragments corresponding to PrP 27–30), which are pathognomonic of prion disease [30]. This classical PrP^Sc^ in 117VV transgenic mouse brain is, however, extremely labile in comparison to classical PrP^Sc^ generated in transgenic mouse models of P102L or E200K [30,59], and such intrinsic instability may explain why classical PrP^Sc^ signatures in A117V patient brain have not been detected.

Notably, as part of these studies, we observed occasional PrP plaques in the brain of uninoculated old-aged 117VV Tg30 mice. Here, we now demonstrate that these pathological changes are associated with spontaneous production of disease-related PrP assemblies having molecular and phenotypic transmission properties that mirror those generated in IPD A117V patient brain.

## Results

### Age-dependent spontaneous formation of abnormal PrP deposits in the brain of aged 117VV Tg30 mice

**Fig 1:**
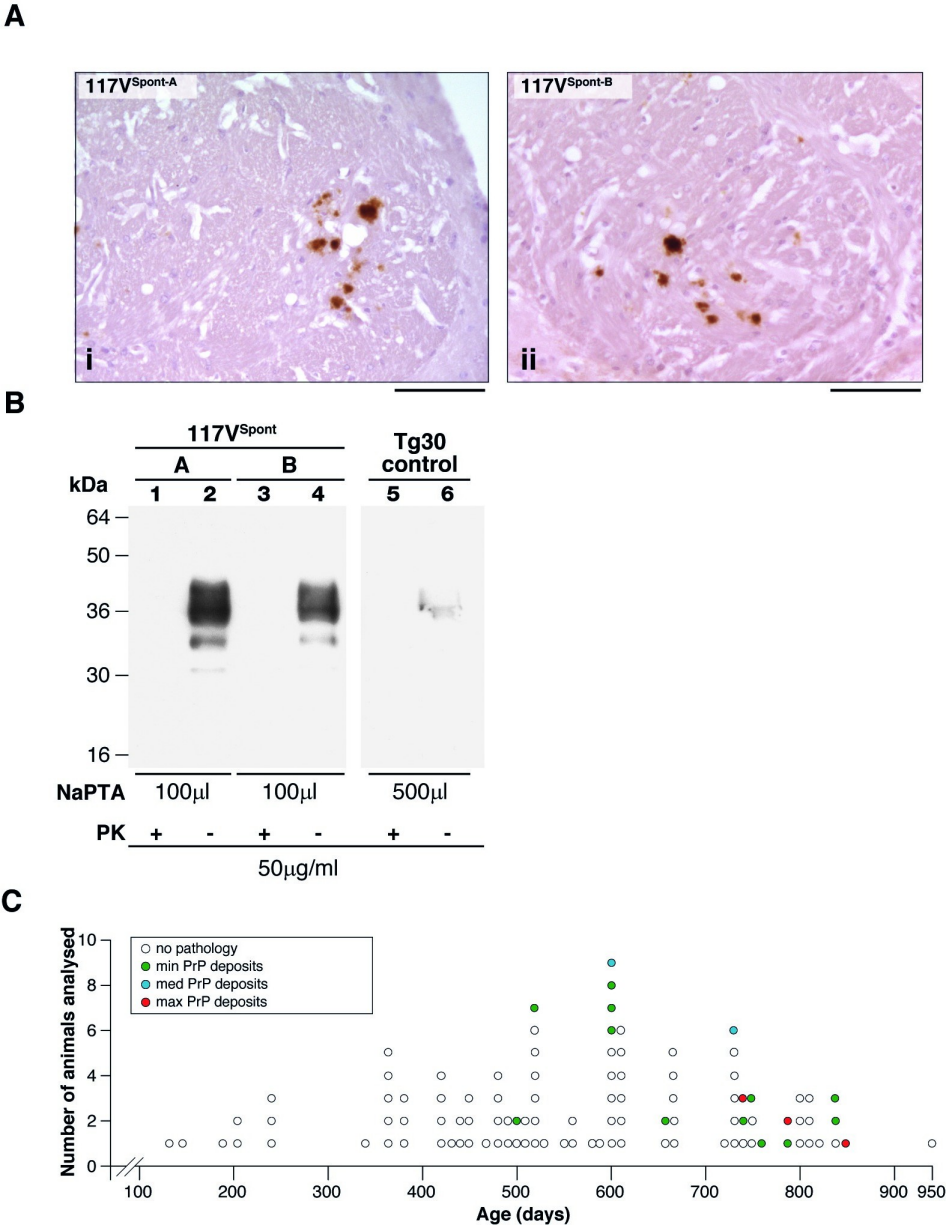
Characterisation of abnormal PrP deposition in the brain of uninoculated 117VV Tg30 transgenic mice. (A, i and ii) IHC using anti-PrP monoclonal antibody 3F4 showing PrP plaques in the anterior commissure of the brain of aged 117VV Tg30 mice. (Ai) 117V^Spont-A^ mouse that developed neurological disease at 476 days of age. (Aii) 117V^Spont-B^ mouse culled at 734 days without neurological symptoms. Scale bar, 300 μm. (B) Immunoblot developed with anti-PrP monoclonal antibody ICSM 35 (epitope human PrP residues 93–105) and high sensitivity chemiluminescence showing insoluble PrP recovered after NaPTA-precipitation of 10% (w/v) brain homogenate. Lanes 1 and 2, 117V^Spont-A^ brain; lanes 3 and 4, 117V^Spont-B^ brain; lanes 5 and 6, age-matched control 117VV Tg30 brain that was devoid of PrP plaques when analysed by IHC. The volumes of 10% (w/v) brain homogenate from which NaPTA pellets were derived are shown in microliters (μL). NaPTA pellets in lanes 1, 3, and 5 were digested with proteinase-K under standard conditions (PK+), and the entire processed sample was loaded on the gel. Samples in lanes 2, 4, and 6 were analysed without PK digestion (PK−) and 20% of the processed NaPTA pellet loaded on the gel because this produced PrP signal intensities that fall within the linear range of the chemiluminescent substrate. (C) Graphical representation of the incidence and severity of spontaneous PrP plaque deposition in brain of uninoculated 117VV Tg30 mice versus age (S1 Data). Brain was examined by IHC using anti-PrP monoclonal antibody ICSM 35. Representative examples of minimum (min), medium (med), and maximum (max) levels of spontaneous 117V PrP deposition in aged uninoculated 117VV Tg30 mice are shown in S1 Fig. This PrP severity scoring only represents what was seen in this aged cohort of 117VV Tg30 mice. ICSM, Imperial College School of Medicine; IHC, immunohistochemistry; NaPTA, sodium phosphotungstic acid; PK, proteinase K; PrP, prion protein.

Transgenic mice homozygous for human PrP 117V (117VV Tg30 mice and 117VV Tg31 mice) are fully susceptible to infection with disease-related PrP assemblies that propagate in the brain of patients with IPD A117V [30]. As part of these studies, we monitored a cohort of 20 uninoculated 117VV Tg30 mice over their lifespan in order to see whether the expression of mutant 117V human PrP might lead to age-dependent development of spontaneous disease. Three mice from this cohort showed symptomatic neurological signs and were culled at ages of 476, 600, and 742 days [30]. Brains from these mice and from other mice in the group (that were culled either because of old age or the occurrence of nonneurological intercurrent illness) were divided sagittally with one half fixed for neuropathological examination and PrP immunohistochemistry (IHC) and the other frozen. We found that the mouse culled with neurological disease at 476 days old (designated 117V^Spont-A^) and another mouse without neurological dysfunction culled at 734 days old (designated 117V^Spont-B^) had immuno-reactive PrP plaques that were localised to the anterior commissure of the brain (Fig 1Ai and 1Aii, respectively). All other brains examined from this cohort of aged 117VV Tg30 mice were devoid of neuropathological changes or abnormal PrP deposition. This restricted pattern of spontaneous PrP deposition, seen in only a proportion of aged 117VV Tg30 mice, differed markedly from the uniform and widespread pattern of PrP deposition seen in all 117VV Tg30 mice following challenge with A117V IPD patient brain [30].

Subsequently, we examined 10% (w/v) homogenate prepared from the frozen, contralateral side of brain from the 117V^Spont-A^ and 117V^Spont-B^ mice for the presence of abnormal PrP using high-sensitivity immunoblotting. Using standard procedures [62], no proteinase K (PK)-resistant PrP could be detected; however, using sodium phosphotungstic acid (NaPTA) precipitation to isolate disease-related PrP aggregates from a larger volume of brain homogenate [7,62,63], we found that both 117V^Spont-A^ and 117V^Spont-B^ mouse brains contained aggregates of insoluble 117V PrP (Fig 1B lanes 2 and 4), although this material was not resistant to digestion with PK using standard conditions (Fig 1B lanes 1 and 3). Importantly, the ability to precipitate PrP with NaPTA appeared to be specific to the 117V^Spont-A^ and 117V^Spont-B^ mouse brains that harboured the spontaneous PrP plaques because we could not detect insoluble PrP following similar analyses of much larger volumes of comparably age-matched 117VV Tg30 brain that were devoid of detectable PrP plaques when examined by IHC (Fig 1B lanes 5 and 6).

Based upon these findings, we initiated 2 experiments. Firstly, we inoculated 117V^Spont-A^ and 117V^Spont-B^ mouse brains into further groups of 117VV Tg30 mice to test for the presence of transmissible PrP assemblies (see below). Secondly, in order to determine the earliest age at which preclinical spontaneous PrP plaque deposits could be detected in 117VV Tg30 mouse brain, we set up a larger cohort of uninoculated mice in which groups were killed at specific time points. Using IHC analyses, we found that the incidence and severity of spontaneous PrP plaque deposition in the brain of 117VV Tg30 mice increased with age, with the first occurrence being at around 500 days (Fig 1C and S1 Data). Spontaneously generated PrP plaques were typically first detected in the anterior commissure, after which other brain areas became affected as the mice aged (S1 Fig). Notably, the occurrence of PrP plaque deposition in the brain of aged 117VV Tg30 mice remained stochastic rather than an inevitable consequence of old age (Fig 1C and S1 Data). In this regard, the oldest mouse was 950 days old when killed and did not display neurological symptoms, and its brain was devoid of abnormal PrP deposits when examined by IHC (Fig 1C and S1 Data).

Importantly, the deposition of abnormal PrP in 117VV Tg30 mouse appears to be directly related to the presence of the 117V point mutation in human PrP rather than overexpression of PrP per se. The 117V mutation in IPD patients is exclusively found on a *PRNP* 129V allele and in contrast to the PrP deposition seen in 117VV Tg30 mouse brain in which mutant PrP overexpression is 2-fold (compared to normal human brain) transgenic mice with 6-fold overexpression of wild-type human PrP 129V (129VV Tg152 or Tg152c mice) have never shown evidence for spontaneous development of PrP plaques in old age (>200 brains examined by IHC) [19,64,65].

### 117VV Tg30 mouse brains with spontaneous PrP deposits contain infectious PrP assemblies with transmission properties that mirror those from IPD A117V patient brain

**Fig 2:**
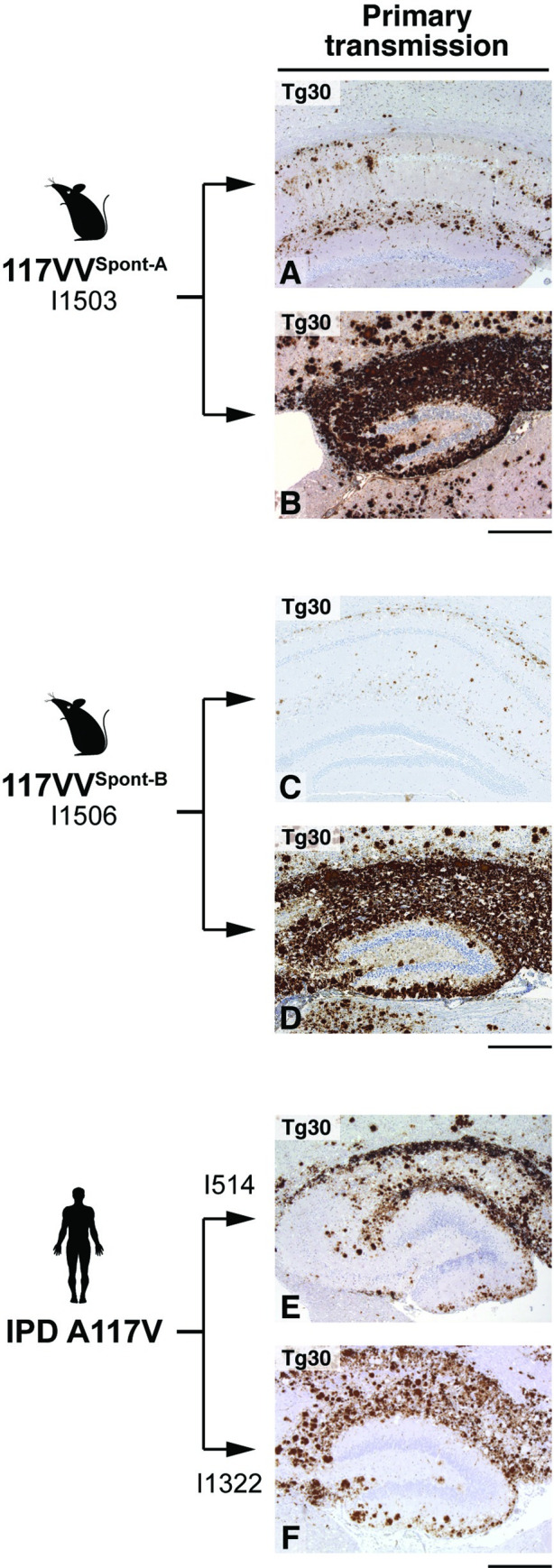
Immunohistochemical analyses of brain from 117VV Tg30 mice challenged with spontaneous 117V prion isolates or IPD A117V patient brain. IHC was performed using anti-PrP monoclonal antibody ICSM 35 (epitope human PrP residues 93–105). All panels show the hippocampus. (A, B) Transmission of homogenate from 117V^Spont-A^ mouse brain. Panel A shows brain from a mouse culled at 273 days post-inoculation without neurological disease and panel B shows the brain from a mouse culled at 733 days post-inoculation with neurological disease. (C, D) Transmission of homogenate from 117V^Spont-B^ mouse brain. Panels C and D show brain from mice culled without neurological disease at 238 and with neurological disease at 616 days post-inoculation, respectively. (E, F) Transmission of two IPD A117V patient brain homogenates (I514 and I1322 [30]). Scale bar, 300 μm. ICSM, Imperial College School of Medicine; IHC, immunohistochemistry; IPD, inherited prion disease; PrP, prion protein.

**Fig 3:**
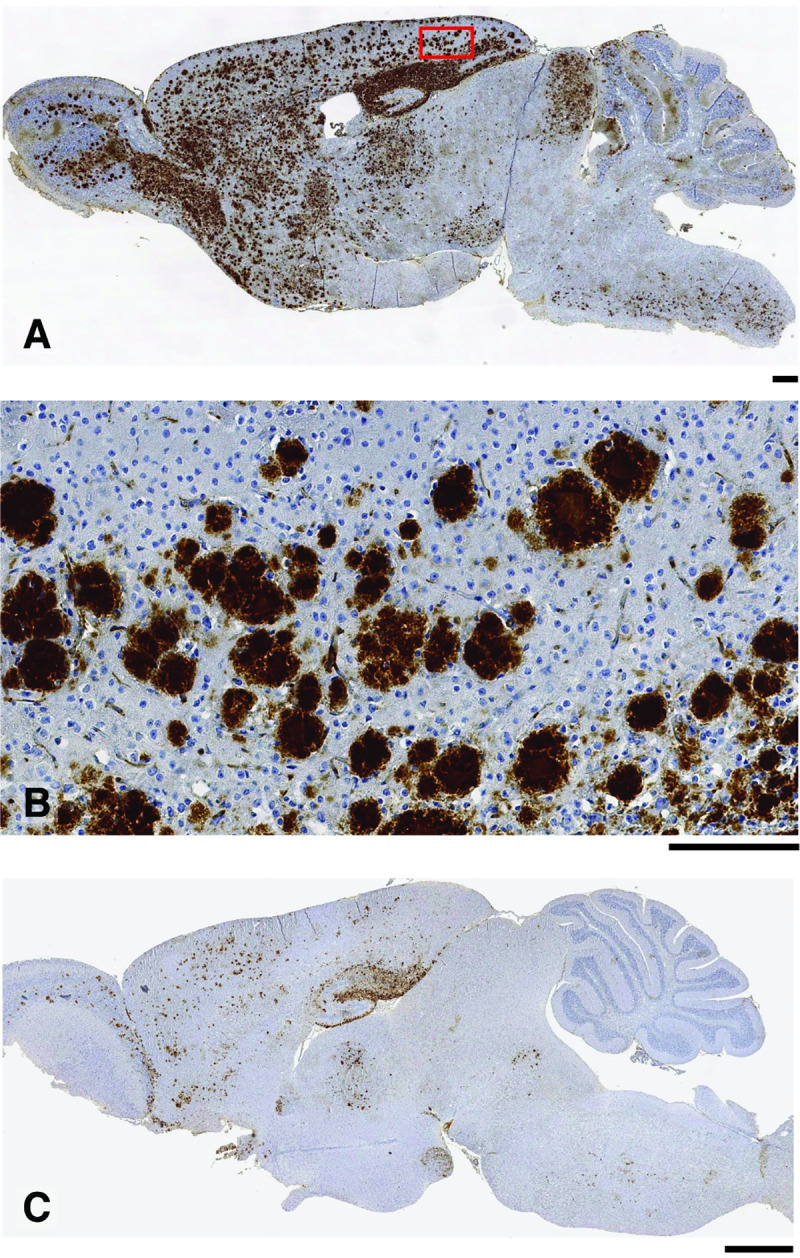
Immunohistochemical analyses of brain from 117VV Tg30 mice challenged with a spontaneous 117V prion isolate. (A, B) Primary transmission of spontaneous 117V prion isolate (from 117V^Spont-A^ mouse brain) to 117VV Tg30 mice. Brain from challenged mice was examined by IHC using anti-PrP monoclonal antibody ICSM 35. (A) Sagittal brain section from a mouse culled at 733 days post-inoculation with neurological disease showing widespread PrP deposition. (B) Magnification of the midsection of the same brain shown in panel A showing detailed granular nature of the PrP plaques. (C) Sagittal brain section from an uninoculated 117VV Tg30 mouse (culled at 852 days old without neurological disease; see Fig 1C and S1 Data) showing the maximum intensity of spontaneous PrP deposition that we observed in aged uninoculated 117VV Tg30 mice. Scale bars: panels A and B, 300 μm; panel C, 1 mm. ICSM, Imperial College School of Medicine; IHC, immunohistochemistry; PrP, prion protein.

Brain homogenates from 117V^Spont-A^ and 117V^Spont-B^ mice were inoculated intracerebrally into further groups of 117VV Tg30 mice that were then monitored for neurological symptoms over their lifespan and brain samples collected at autopsy to study abnormal PrP deposition. Remarkably, we found that all mice challenged with either the 117V^Spont-A^ or 117V^Spont-B^ brain homogenate became infected, resulting in intense and widespread PrP plaque deposition throughout the brain (Table 1, Fig 2, S2 Fig, and Fig 3).

**Table 1:** Primary transmission of spontaneous 117V prion isolates and IPD A117V patient brain to 117VV Tg30 mice.

Type of brain inoculum	Code	117VV Tg30			
		Total attack rate^1^	Clinically affected	Incubation period (days p.i.)^2^	Subclinically infected
117V^Spont-A^	I1503	9/9	2/9	566, 733	7/9^3^
117V^Spont-B^	I1506	6/6	2/6	616, 616	4/6^4^
IPD A117V^5^	I514	5/5	0/5	>400–528	5/5
	I1321	5/5	0/5	>461–828	5/5
	I1322	4/4	0/4	>327–612	4/4

^1^Total Attack rate is defined as the total of clinically affected and subclinically infected mice as a proportion of the number of inoculated mice. Subclinical prion infection was assessed by IB and/or IHC examination of brain.

^2^Incubation periods are reported for clinically affected mice in days p.i. When no clinical transmission of prion disease was observed, the interval between inoculation and death (from culling because of senescence, intercurrent illness, or termination of the experiment) is reported as >x–y days.

^3^Mice with subclinical infection were culled at 273, 404, 588, 657, 658, 728, and 728 days p.i.

^4^Mice with subclinical infection were culled at 238, 283, 352, and 525 days p.i.

^5^Primary transmission of IPD A117V prions from patient brain has been previously published [30].

**Abbreviations:** IB, immunoblotting; p.i., post-inoculation; IHC, immunohistochemistry; IPD, inherited prion disease

Neuronal loss and spongiosis was also observed, the intensity of which appeared to be related to the PrP plaque load in the affected brain area (S3 Fig). However, in the majority of mice (11/15), this infection did not produce neurological symptoms despite the severity of the PrP plaque deposition (Table 1). Notably, some of the subclinically infected mice (4/11) that showed abundant PrP plaque deposition in multiple brain areas were culled when less than 409 days old (Table 1). This finding contrasts strikingly with the complete absence of spontaneous PrP plaques in comparably aged uninoculated 117VV Tg30 (Fig 1C and S1 Data). Collectively, these transmission data establish that spontaneous generation of infectious PrP assemblies has occurred in the brain of the 117V^Spont-A^ and 117V^Spont-B^ mice. To facilitate readability, we hereafter refer to these as spontaneous 117V prion isolates.

**Fig 4:**
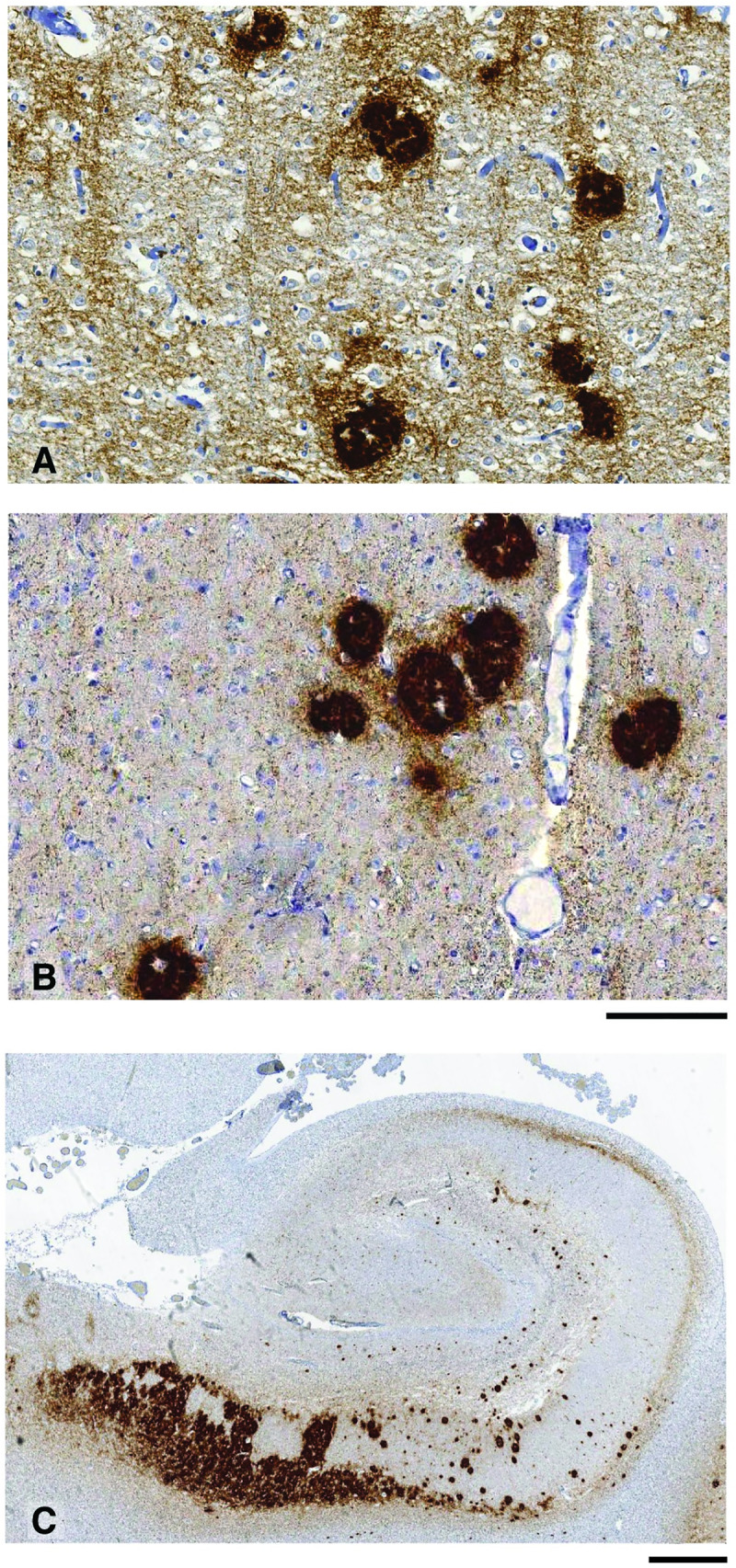
Immunohistochemical analyses of brain from an IPD A117V patient. IHC was performed using anti-PrP monoclonal antibody ICSM 35 (epitope human PrP residues 93–105). Panels A, B, and C show the frontal cortex, thalamus, and hippocampus, respectively, from IPD A117V patient brain collected at autopsy. The morphology of PrP plaques is closely similar to those seen in 117V Tg30 mouse brain after challenge with either spontaneous 117V prion isolates or IPD A117V patient brain. Scale bar: panels A and B, 100 μm; panel C, 1 mm. ICSM, Imperial College School of Medicine; IHC, immunohistochemistry; IPD, inherited prion disease; PrP, prion protein.

**Fig 5:**
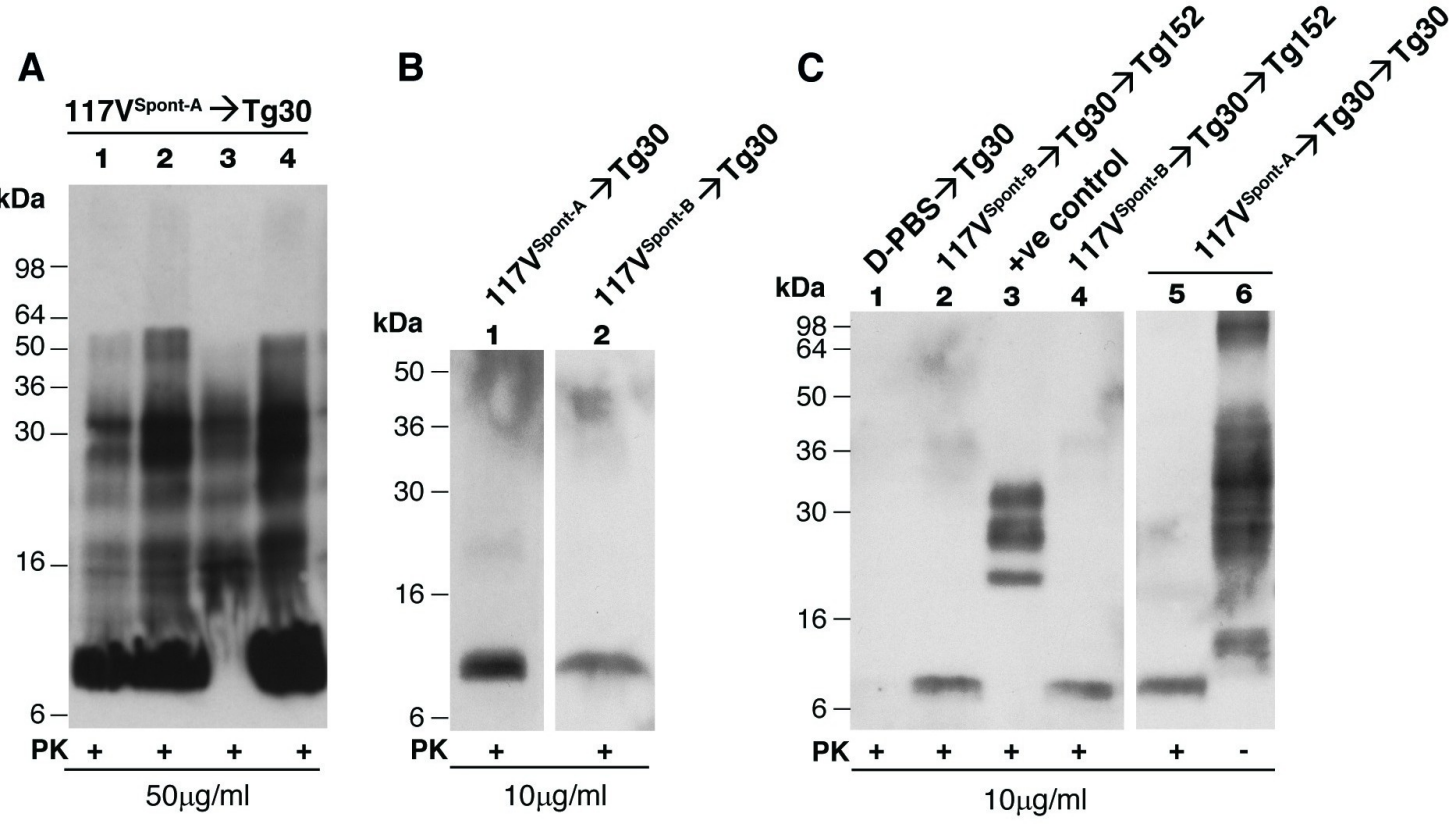
Immunoblot detection of disease-related PrP in transgenic mouse brain. Panels A–C show transgenic mouse brain homogenates (10% (w/v) in D-PBS) analysed with anti-PrP monoclonal antibody ICSM 35 (epitope human PrP residues 93–105) and high-sensitivity chemiluminescence either before (−) or after (+) digestion with PK. The provenance of each brain sample is designated above each lane. Brain homogenates analysed in panel A underwent only 1 freeze/thaw cycle prior to analysis whereas those in panels B and C underwent 2 freeze/thaw cycles prior to analysis. (A) Primary transmission of spontaneous 117V prion isolate from 117V^Spont-A^ mouse brain to 117VV Tg30 mice. Classical proteolytic fragments of PrP^Sc^ (akin to PrP 27–30; lanes 1–4) and an 8 kDa PrP fragment (together with possible multimers thereof migrating at 16–18 and 22–23 kDa [32]; lanes 1, 2, and 4) are seen in brain homogenates that have not undergone repetitive freeze/thaw cycles prior to PK digestion. This pattern of disease-related PrP fragments is closely similar to the pattern seen in the brain of 117VV mice following primary transmission of IPD A117V prions from patient brain [30]. (B) Primary transmission of spontaneous 117V prion isolates from 117V^Spont-A^ and 117V^Spont-B^ mouse brain to 117VV Tg30 mice. Brain homogenates that underwent repetitive freeze/thaw cycles prior to digestion with PK show only an 8 kDa PK-resistant PrP fragment. (C) Secondary transmission of spontaneous 117V prion isolates. Spontaneous 117V prion isolates from 117V^Spont-A^ and 117V^Spont-B^ mouse brains were passaged once in 117VV Tg30 mice and brain homogenates from these transmissions used to inoculate further 117VV Tg30 mice or 129VV Tg152 mice. Subclinically affected mice from these secondary transmissions show an 8 kDa protease-resistant PrP fragment in brain homogenates that underwent repetitive freeze/thaw cycles prior to PK-digestion (lanes 2, 4, and 5). Lane 1 shows brain homogenate from a negative control 117VV Tg30 mouse inoculated with D-PBS. Lane 3 shows brain homogenate from a positive control 129MM Tg35 mouse propagating sporadic CJD prions, and lane 6 shows analysis of the same brain as lane 5 in the absence of PK digestion. CJD, Creutzfeldt-Jakob disease; ICSM, Imperial College School of Medicine; IPD, inherited prion disease; PK, proteinase K; PrP, prion protein.

Subsequent comparative analyses revealed that the transmission properties of spontaneous 117V prion isolates in 117VV Tg30 mice mirrored the transmission properties of brain homogenates from IPD A117V patients in human PrP 117V expressing mice (Table 1) [30]. The similarity between the patterns of PrP plaque deposition in the brain of 117VV Tg30 recipients challenged with spontaneous 117V prion isolates or IPD A117V patient brain is illustrated in Fig 2. Indeed, this distinctive PrP plaque morphology is also directly seen in IPD A117V patient brain (Fig 4). Importantly, immunoblot analyses of 117VV Tg30 mouse brain showed that recipients challenged with spontaneous 117V prion isolates also propagated abnormal PrP assemblies that were seen after challenge of 117VV Tg30 and 117VV Tg31 mice with IPD A117V patient brain homogenate (Fig 5) [30]. These comprise a labile PrP^Sc^ assembly generating proteolytic fragments of molecular mass 21 to 30 kDa (equivalent to prototypical PrP 27–30) [30] and an alternative and more stable PrP assembly that generates a characteristic proteolytic fragment of 8 kDa (Fig 5) [30] that can also form stable multimers migrating at 16 to 18 kDa and 22 to 23 kDa [32]. Collectively, the striking similarity of the molecular and neuropathological phenotypes that we observed strongly suggests that infectious assemblies of human PrP 117V that were spontaneously generated in the brain of 117VV Tg30 mice are congruent with those generated in IPD A117V patient brain.

### Transmission properties of spontaneous 117V prion isolates after secondary passage in 117VV Tg30 mice

**Fig 6:**
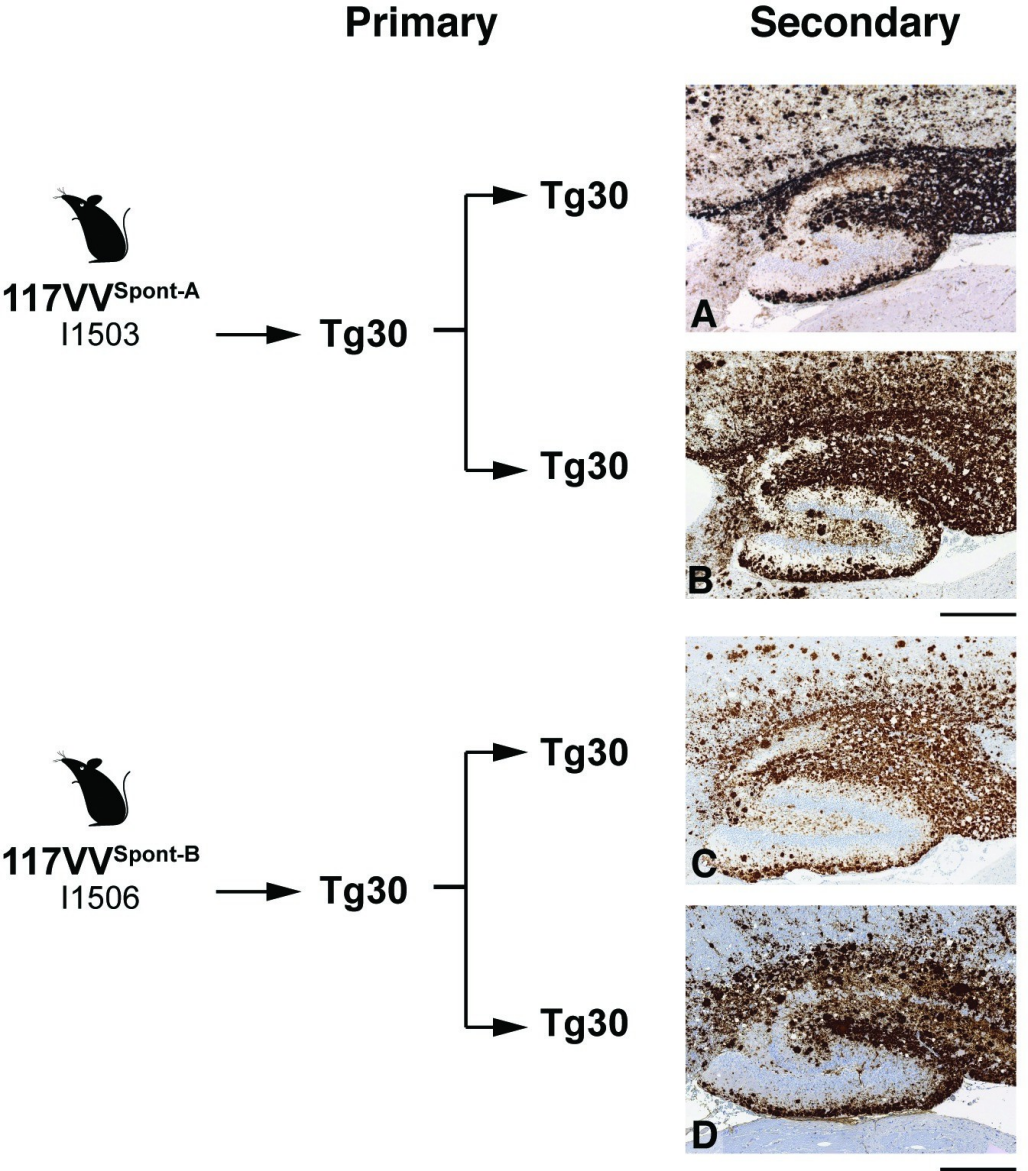
Immunohistochemical analyses of brain from 117VV Tg30 mice challenged with spontaneous 117V prion isolates passaged once in 117VV Tg30 mice. IHC was performed using anti-PrP monoclonal antibody ICSM 35 (epitope human PrP residues 93–105). Panels A–D show the hippocampus. (A, B) Spontaneous 117V prion isolate from 117V^Spont-A^ mouse brain was passaged once in 117VV Tg30 mice and then passaged in further 117VV Tg30 mice. (C, D) Spontaneous 117V prion isolate from 117V^Spont-B^ mouse brain was passaged once in 117VV Tg30 mice and then passaged in further 117VV Tg30 mice. Mice in panels A–D were culled without neurological disease at 455, 457, 417, and 456 days post-inoculation, respectively. Scale bar, 300 μm. ICSM, Imperial College School of Medicine; IHC, immunohistochemistry; PrP, prion protein.

**Fig 7:**
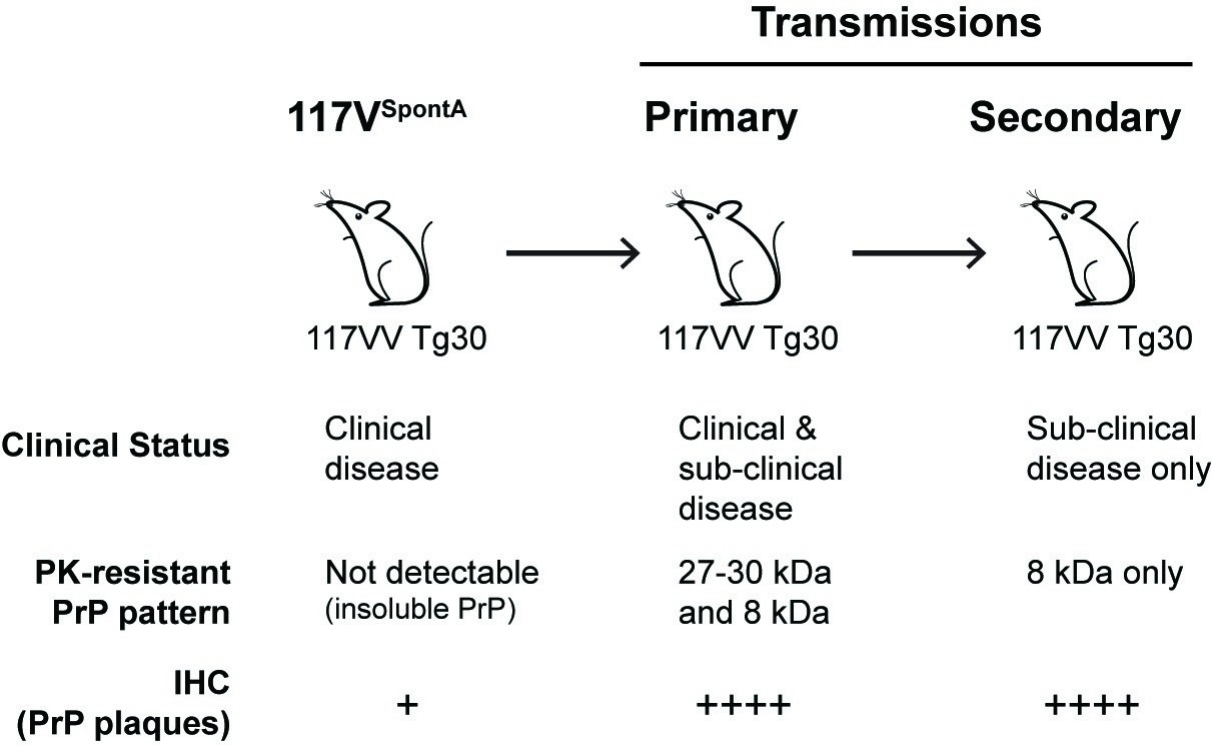
Summary of serial transmission of Spont^A^ mouse brain in further 117VV Tg30 mice. Spont^A^ mouse had clinical prion disease and was culled at 476 days old; focal PrP plaques were found in the anterior commissure of brain, and insoluble PrP aggregates were found in brain homogenate that was not resistant to PK digestion under standard conditions. Primary transmission of Spont^A^ mouse brain to further 117VV Tg30 mice resulted in both clinical neurological and subclinical infection; extensive PrP plaque deposition was found throughout the brain and brain homogenate showed the propagation of distinct abnormal PrP assemblies generating either 27 to 30 kDa or 8 kDa PK-resistant PrP fragments. After secondary transmission in 117VV Tg30 mice extensive PrP plaque deposition throughout the brain was maintained together with the detection of the abnormal PrP assembly generating the 8 kDa PK-resistant PrP fragment; however, all mice remained subclinically affected. IHC, immunohistochemistry; PK, proteinase K; PrP, prion protein.

Spontaneous 117V prion isolates showed no major alteration of their transmission properties after secondary passage in 117VV Tg30 mice (S2 Fig; Table 2, Fig 6, S3 Fig, and S4 Fig). A total of 46 out of 47 inoculated mice were infected (Table 2) with preservation of a neuropathological phenotype dominated by the widespread occurrence of 117V PrP plaques (Fig 6). Notably, all mice in the secondary transmission series were subclinically infected; however, because they were culled before 460 days post-inoculation, it is clearly possible that some of these mice may have developed clinical prion disease had they lived longer. Nevertheless, the secondary passage data firmly establish that spontaneous 117V prion isolates do not adapt to produce a uniformly lethal spongiform encephalopathy with short mean incubation periods following serial passage in 117VV Tg30 mice (see Fig 7, which summarises the serial transmission findings for 117V^Spont-A^ mouse brain).

**Table 2:** Secondary passage of spontaneous 117V prion isolates in 117VV Tg30 and 129VV Tg152 mice.

Inoculum details^1^	Code	117VV Tg30					129VV Tg152				
		Total attack rate^2^	Clinical attack rate	Incubation period^2^ (days p.i.)	IB	IHC	Total attack rate^2^	Clinical attack rate	Incubation period^2^ (days p.i.)	IB	IHC
Passaged 117V Spont^-A^	I3912	8/8	0/8	>211–456	8/8	7/7	0/6	0/6	>185–457	0/6	0/6
	I3911	8/8	0/8	>417–457	7/8	8/8	6/8	0/8	>427–456	0/8	6/8
	I4653	8/8	0/8	>238–454	8/8	8/8	0/8	0/8	>307–456	0/8	0/8
	Total	24/24	0/24		23/24	23/23	6/22	0/22		0/22	6/22
Passaged 117V Spont^-B^	I3913	5/5	0/5	>416	5/5	5/5	4/9	0/9	>268–457	4/9	0/9
	I3914	9/10	0/10	>259–456	9/9	9/10	1/6	0/6	>346–456	1/6	0/6
	I4654	8/8	0/8	>197–456	4/8	8/8	0/7	0/7	>355–456	0/7	0/7
	Total	22/23	0/23		18/22	22/23	5/22	0/22		5/22	0/22

^1^Spontaneous 117V prion isolates from Spont^-A^ and Spont^-B^ 117VV Tg30 mouse brain were passaged once in further 117VV Tg30 mice (see Table 1, S2 Fig). Provenance of the mice from which 1% (w/v) brain inocula were prepared are provided in S2 Fig.

^2^Total attack rate is defined as the total of clinically affected and subclinically infected mice as a proportion of the number of inoculated mice. Subclinical prion infection was assessed by IB and/or IHC examination of brain. Because no clinical transmission of prion disease was observed, the interval between inoculation and death (from culling because of intercurrent illness or termination of the experiment) is reported as >x–y days.

**Abbreviations:** IB, immunoblotting; IHC, immunohistochemistry; p.i., post-inoculation

One possible explanation for this apparent lack of adaptation may relate to the relative efficiencies of replication of the 2 disease-related 117V PrP assemblies that are propagating in these transmissions. Although accurate measurement of the concentration of the stable 117V PrP amyloid assembly would be possible, the inherent instability of the labile 117V PrP^Sc^ assembly [30] precludes reliable determination of its concentration using currently available methods. Consequently, it has not been possible to determine whether there is a difference in the ratio of the 2 disease-related 117V PrP assemblies in the brain of aged-matched clinically affected or subclinically infected 117VV Tg30 mice. However, in the case of the clinically affected 117V^Spont-A^ mouse, its brain showed only focal 117V PrP plaques at a level very much lower than present in the brain of subclinically infected mice from the primary or secondary transmission series. These observations suggest that 117V PrP plaque load is dissociated from a clinical phenotype. Preferential propagation of the stable 117V PrP amyloid assembly rather than the labile 117V PrP^Sc^ assembly may therefore result in a progressive clinically silent PrP amyloidosis. Testing the validity of this hypothesis and whether efficient propagation of labile 117V PrP^Sc^ is responsible for clinical phenotype will now require the development of new methods for isolation of each distinct 117V PrP assembly.

### Spontaneously generated 117V isolates can infect transgenic mice expressing wild-type human PrP

Variable conversion of wild-type PrP to pathogenic conformations is thought to be an important contributor to phenotypic variability in certain IPDs associated with different mutations [28,29,66–68]. Co-propagation of distinct disease-related PrP assemblies (infectious paired fibre PrP rods composed of proteotypical PrP^Sc^ or distinct PrP amyloid fibres) generated from either mutant or wild-type PrP, combined with differences in their regional distribution, abundance, and potential neurotoxicity, provides a complex molecular mechanism for generating phenotypic heterogeneity in patients with the same *PRNP* mutation.

**Fig 8:**
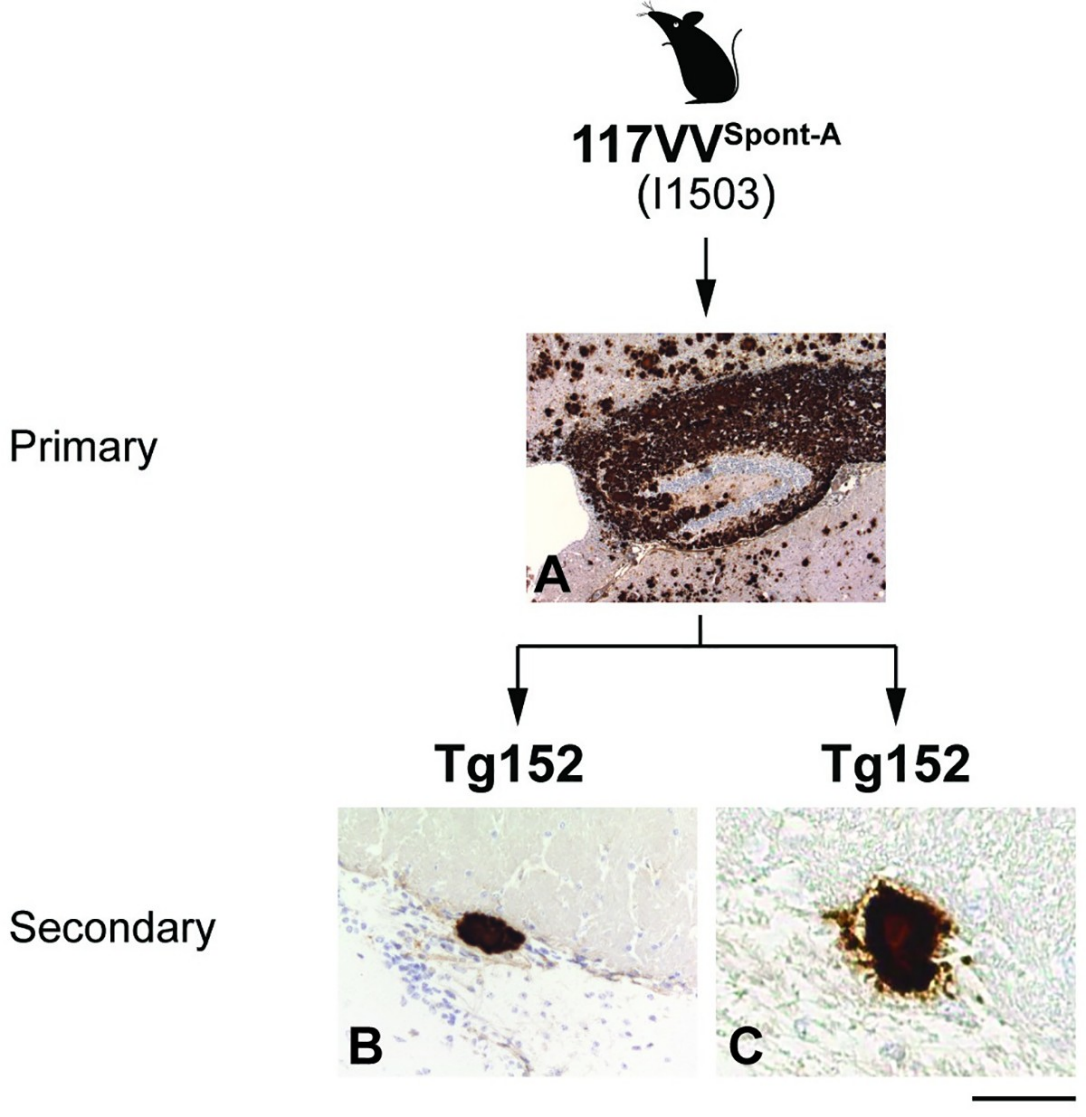
Immunohistochemical analyses of brain from 129VV Tg152 mice challenged with a spontaneous 117V prion isolate passaged once in 117VV Tg30 mice. IHC was performed using anti-PrP monoclonal antibody ICSM 35 (epitope human PrP residues 93–105). Panel A shows the hippocampus; panels B and C show the corpus callosum. (A) Primary transmission of a spontaneous 117V prion isolate (from 117V^Spont-A^ mouse brain) to 117VV Tg30 mice. (B, C) Spontaneous 117V prion isolate from 117V^Spont-A^ mouse brain was passaged once in 117VV Tg30 mice and then passaged in 129VV Tg152 mice. The mouse in panel A was culled with clinical disease at 733 days post-inoculation, and the mice in panels B and C were culled without neurological disease at 456 days post-inoculation. Scale bar, 300 μm. ICSM, Imperial College School of Medicine; IHC, immunohistochemistry; PrP, prion protein.

Accordingly, in further transmission experiments, we examined whether spontaneous 117V prion isolates passaged once in 117VV Tg30 mice could also propagate in transgenic mice expressing wild-type human PrP 129V (S2 Fig). Surprisingly, we found that 25% (11/44) of inoculated 129VV Tg152 mice became subclinically infected. Abnormal PrP deposition, where detectable, was restricted to occasional, small PrP plaques in the corpus callosum (Fig 8), and immunoblot analysis showed the propagation of a disease-related wild-type PrP assembly that generated a proteolytic fragment of 8 kDa closely similar to that seen in 117VV Tg30 mice following challenge with the same isolates (Fig 5). In our wide experience of transmitting multiple sporadic or acquired CJD isolates to 129VV Tg152 mice [19,20,69–72] or the congenic 129VV Tg152c version of this line [65,73], we have never observed the propagation of a disease-related PrP isoform that generates a proteolytic fragment of 8 kDa rather than the typical PrP 27–30 fragment pattern derived from prototypical PrP^Sc^. These data suggest that the stable 117V PrP assembly that generates the 8 kDa proteolytic fragment and forms amyloid plaques in 117VV Tg30 mice can also template the misfolding of wild-type human PrP 129V to a similar amyloid conformation. Based upon these findings, primary transmissions of A117V patient brain to mice expressing wild-type human PrP should be revisited and compared to the transmission properties of the same A117V patient isolates after passage in 117VV Tg30 mice to see if the transmission frequency of clinically silent amyloid phenotypes is similar.

### Transgenic modelling provides insight into disease pathogenesis in IPD A117V patients

Spontaneous formation of disease-related PrP assemblies in transgenic mice expressing human PrP 117V but not in transgenic mice expressing human PrP 102L [43,59] or human PrP 200K [59] suggests that the spontaneous formation of abnormal 117V PrP assemblies occurs on a faster time scale. To seek evidence for this in humans, we examined the age of clinical disease onset in IPD patients with these different *PRNP* point mutations. We found that the mean age of clinical onset in IPD A117V patients was significantly earlier than seen in either IPD P102L or E200K patients (Table 3). These data suggest that the ability to model spontaneous prion disease pathogenesis in transgenic mice may be restricted to pathogenic human PrP mutants that are associated with early onset of disease. In this regard, it is likely that overexpression of mutant PrP^C^ in the transgenic mice, coupled with inherent faster replication kinetics of 117V disease-related PrP assemblies would have acted together to result in the evolution of disease pathogenesis within the relatively short lifespan of a mouse.

**Table 3:** Age at clinical onset, duration and difference between IPD codon 129 homozygotes and heterozygotes.

*PRNP* mutation^1^	Mean AAO (years ± standard deviation)^2^	Mean clinical duration (months)	Mean difference in AAO between codon 129 homozygotes and heterozygotes (years)	Total no. of patients
A117V	39.2 ± 7.0	49.4	−10.0^3^	19
P102L	49.1 ± 10.8	48.0	−5.8^4^	38
E200K	61.1 ± 11.6	5.1	1.3^5^	18

^1^Patients with IPD were identified according to established WHO criteria by the NPC, London, the National CJD Research and Surveillance Unit, Edinburgh, and other referrers in the United Kingdom in the period from 1995 to 2015. For patients with the P102L mutation in *PRNP*, all 38 were assessed by the NPC and/or reported by Webb and colleagues [40]. For patients with the E200K mutation in *PRNP*, all 18 patients were seen by the NPC and recruited in the National Prion Monitoring Cohort study [87]. For patients with the A117V mutation in *PRNP*, 11 were assessed by the NPC and/or reported by Mallucci and colleagues [61], and 8 further patients were reported by European colleagues [25,88,89].

^2^All pairwise comparisons between mutations for mean AAOs were statistically significant (independent samples t-test, A117V versus P102L, *p* = 0.01; A117V versus E200K, *p* = 6.4 × 10^−7^; P102L versus E200K, *p* = 7.5 × 10^−4^). The mean duration was much shorter for E200K compared to either P102L or A117V (independent samples *t* test, A117V versus E200K, *p* = 1.3 × 10^−7^).

^3^The AAO for A117V valine homozygous individuals (*n* = 6) was 10.0 years earlier than for methionine-valine heterozygous individuals (*n* = 13), and this difference was statistically significant (independent samples *t* test, A117V codon 129 homozygous versus heterozygous, *p* = 0.039).

^4^The AAO for P102L codon 129 homozygous versus heterozygous was not statistically significant in these 38 patients and was significantly different in a previously reported pedigree [40] but not in a large international series [90].

^5^The AAO for E200K codon 129 homozygous versus heterozygous was not statistically significant.

**Abbreviations:** AAO, Age at clinical onset; IPD, inherited prion disease; NPC, National Prion Clinic; *PRNP*, human prion protein gene

Our finding that spontaneous 117V prion isolates once amplified in 117VV Tg30 mice can convert wild-type human PrP 129V to a protease-resistant form suggests that the pathological involvement of wild-type PrP may be an important modifier of disease pathogenesis in IPD A117V patients. Given the profound effect that codon 129 mismatch in wild-type PrP has in controlling disease pathogenesis in sporadic and acquired CJD (where heterozygosity at codon 129 is thought to confer resistance to prion disease by inhibiting homologous protein-protein interactions [71,74–76] and the presence of 129M or 129V controls the propagation of distinct human prion strains via conformational selection [2,9,15,18–20]), we looked for similar effects in IPD A117V patients. In keeping with similar findings with IPD associated with an insertion of 4, 5, or 6 extra octapeptide repeats [77–79], or in a pedigree of IPD P102L [40,80], we found that the age of onset of disease in IPD A117V patients whose wild-type PrP codon 129 genotype matched that of the mutant 117V PrP (129V) was significantly shorter than in codon 129 heterozygous patients (Table 3). The demonstration of conversion of wild-type PrP 129V in transgenic mice following challenge with infectious 117V PrP assemblies now provides a rational molecular basis for this effect.

## Discussion

The extraordinary heterogeneity of neurodegenerative disease clinical phenotypes remains poorly understood. Study of human and animal prion diseases has led to a hypothesis that “strains” of disease-associated misfolded protein determine the brain regions most involved, the intensity of neurotoxicity, and hence clinical phenotype. In this study, we have developed an authentic animal model of IPD, which during aging spontaneously generates abnormal PrP assemblies and shows similar transmission properties in 117VV Tg30 mice compared to brain samples from IPD A117V patients. At the molecular level, these transmissions show clear evidence for the propagation of 2 distinct disease-related 117V PrP assemblies. These comprise a labile PrP^Sc^ assembly generating proteolytic fragments equivalent to prototypical PrP 27–30 similar to those typically seen in CJD, and a more stable PrP assembly that generates a characteristic proteolytic fragment of 8 kDa typically seen in GSS [30,31,32]. Efficient propagation of the labile PrP^Sc^ assembly is hypothesised to correlate with the occurrence of clinical prion disease whereas propagation of the stable amyloid PrP assembly appears to be tolerated by mice. At present, the structural similarity between the labile 117V PrP^Sc^ and classical PrP^Sc^ formed from wild-type PrP remains to be determined.

These findings are consistent with the highly distinct transmission properties of similar disease-associated PrP assembly states from GSS patients with the P102L *PRNP* mutation [33–35] and strongly support our recent proposal that authentic prions (infectious PrP rods 20 nm in width composed of prototypical PrP^Sc^) are distinct from amyloid PrP assemblies that characterise the GSS IPD neuropathological phenotype [37,38].

To the best of our knowledge, 117VV Tg30 mice represent the first authentic transgenic model capable of recapitulating spontaneous prion disease pathogenesis seen in IPD, and these findings significantly support the protein-only hypothesis of prion propagation. Although other models that have superimposed human mutations onto rodent PrP are also capable of spontaneous formation of infectious PrP assemblies [44–54], our experience of direct modelling with mutant human PrP primary sequences [20,43,59] suggests that models involving mutated rodent PrP generate novel experimental prion strains rather than recapitulating the strains causing the human diseases.

The spontaneous generation of infectious 117V PrP assemblies in transgenic mice that we describe here provides important new insight into disease pathogenesis in IPD A117V patients. As our previous transgenic models of IPD P102L and E200K failed to generate spontaneous abnormal PrP assemblies, we speculated that the kinetics of spontaneous PrP misfolding associated with the A117V mutation may be more rapid, thereby enabling observation of de novo initiation of prion pathogenesis within the lifespan of a mouse. Comparison of the mean ages of clinical disease onset in patients strongly supports this idea, because IPD A117V patients have a significantly earlier age of onset than patients with IPD P102L or E200K. Moreover, the significant effect of codon 129 zygosity in modulating the age of disease onset seen in IPD A117V patients can now be reasonably explained by our finding that spontaneously generated 117V infectious PrP assemblies can convert wild-type human PrP 129V to a protease-resistant disease-related PrP assembly. However, it is important to mention that involvement of an additional mechanism for this effect cannot be excluded, because we have not yet examined whether wild-type human PrP 129M has partial dominant-negative inhibitory effects [73,76,81,82] on propagation of infectious PrP assemblies from mutant PrP 117V.

## Conclusions

In summary, we report the first model of IPD that spontaneously generates both human prions and PrP amyloid. Our demonstration that 117VV Tg30 mice faithfully recapitulate IPD A117V molecular and neuropathological phenotypes suggests that these mice may be useful for future translational studies. Comparison of treatment groups with untreated aged cohorts of 117VV Tg30 mice should have the statistical power to inform upon the early or presymptomatic pathogenesis of disease and efficacy of long-term treatments that may prevent spontaneous prion disease. Treatments have or are being developed that target normal PrP [83–85], although these will need to have demonstrable safety before they could reasonably be tested in prevention of disease in healthy carriers of IPD mutations. The fact that distinct disease-associated PrP assemblies can be generated spontaneously in the same mouse is further evidence to caution against a therapeutic strategy based on targeting of one strain of abnormal PrP assembly [9].

## Methods and materials

### Ethics statement

Storage and biochemical analyses of post mortem human brain samples and transmission studies to mice were performed with written informed consent from patients with capacity to give consent. When patients were unable to give informed consent, assent was obtained from their relatives in accordance with UK legislation and Codes of Practice. Samples were stored and used in accordance with the Human Tissue Authority Codes of Practice and in line with the requirements of the Human Tissue Authority licence held by UCL Institute of Neurology. This study was performed with approval from the National Hospital for Neurology and Neurosurgery and the UCL Institute of Neurology Joint Research Ethics Committee (now National Research Ethics Service Committee, London–Queen Square)—REC references: 03/N036, 03/N038 and 03/N133. Work with mice was performed under approval and licence granted by the UK Home Office (Animals (Scientific Procedures) Act 1986); Project Licence number 70/6454, which conformed to University College London institutional and ARRIVE guidelines (www.nc3rs.org.uk/ARRIVE/).

### Method details

#### Transgenic mice

Transgenic mice homozygous for a human PrP^117V,129V^ transgene array and murine PrP null alleles (*Prnp*^*o/o*^) designated Tg(HuPrP^117V 129V+/+^ *Prnp*^*o/o*^)-30 mice (117VV Tg30) have been described previously [30] and were used without modification. Similarly, transgenic mice homozygous for a wild-type human PrP^129V^ transgene array and murine PrP null alleles (*Prnp*^*o/o*^) designated Tg(HuPrP^129V+/+^ *Prnp*^*o/o*^)-152 mice (129VV Tg152) have been described previously [19,20,69–72] and were used without modification.

### Transmission studies

Strict bio-safety protocols were followed. Inocula were prepared, using disposable equipment for each inoculum, in a microbiological containment level 3 laboratory and inoculations performed within a class 1 microbiological safety cabinet. For primary transmission and serial passages, groups of animals comprising 117VV Tg30 and 129VV Tg152 mice were inoculated with appropriate mouse brain homogenates. Details of primary transmissions of A117V patient brain samples to 117V Tg30 mice are described in the work by Asante and colleagues [30]. The genotype of each mouse was confirmed by PCR of ear tissue DNA prior to inclusion, and all mice were uniquely identified by subcutaneous transponders. Disposable cages were used, and all cage lids and water bottles were also uniquely identified by transponder and remained with each cage of mice throughout the incubation period. Mice (males and females aged 6–8 weeks allocated randomly to experimental groups) were anaesthetised with a mixture of halothane and O_2_ and intracerebrally inoculated into the right parietal lobe with 30 μl of 1% (w/v) brain homogenate prepared in Dulbecco’s phosphate buffered saline lacking Ca^2+^ and Mg^2+^ ions (D-PBS). All mice were then examined daily for early indicators of clinical prion disease, including piloerection, sustained erect ears, intermittent generalised tremor, unsustained hunched posture, rigid tail, mild loss of coordination, and clasping hind legs when lifted by the tail. Definite diagnosis of clinical prion disease (triggering experimental end point) was reached if mice exhibited any two early indicator signs in addition to one confirmatory sign or any two confirmatory signs. The confirmatory signs included ataxia, impairment of righting reflex, dragging of hind limbs, sustained hunched posture, or significant abnormal breathing. Mice were killed (by CO_2_ asphyxiation) if they exhibited any signs of distress or once a diagnosis of prion disease was established. Brains from inoculated mice were removed at autopsy, divided sagittally with half frozen, and half fixed in 10% buffered formol saline. Subsequent immunohistochemical or biochemical investigations were performed blind to sample provenance.

### Neuropathological analysis

Brains were fixed in 10% buffered formalin for at least 48 hours and then immersed in 98% formic acid for 1 hour, postfixed in formalin, and then processed for paraffin wax embedding. Serial sections of 4-μm thickness were pretreated by boiling for 10 minutes in a low ionic strength buffer (2.1 mM Tris, 1.3 mM EDTA, 1.1 mM sodium citrate [pH 7.8]) before exposure to 98% formic acid for 5 minutes. Abnormal PrP accumulation was examined using anti-PrP monoclonal antibodies 3F4 [86] or ICSM 35 (D-Gen Ltd, London) on a Ventana benchmark XT automated immunohistochemical staining machine using proprietary secondary detection reagents (Roche, Burgess Hill, UK) before development with 3'3 diaminobenzedine tetrachloride as the chromogen [62]. Harris haematoxylin and eosin staining was done by conventional methods. Astrogliosis was determined by glial fibrillary acidic protein immunostaining following standard protocol. Appropriate controls were used throughout. Histological slides were digitised on a LEICA SCN400F scanner (LEICA Milton Keynes, UK) at 40× magnification with 65% image compression setting during export. Slides were archived and managed on LEICA Slidepath (LEICA Milton Keynes, UK). For the preparation of light microscopy images, image captures were taken and composed in Adobe Photoshop.

### Immunoblotting

Preparation of brain homogenates (10% (w/v) in D-PBS), PK digestion (standard condition, 50 μg/mL PK in the sample for 1 hour at 37°C), and subsequent immunoblotting using high-sensitivity chemiluminescence was performed as described previously [62,63]. NaPTA precipitation of PrP^Sc^ from 10% (w/v) brain homogenates (117V^Spont-A^ and 117V^Spont-B^ isolates only) was performed using established procedures [62,63]. Blots were probed with anti-PrP monoclonal antibody ICSM 35 (epitope human PrP residues 93–105; D-Gen Ltd, London) in conjunction with an anti-mouse IgG-alkaline phosphatase conjugate and development in chemiluminescent substrate (CDP-Star; Tropix Inc) [62,63]. Primary screening of brain homogenates was performed blind to sample identity.

## Supporting information

S1 Fig Representative examples of minimum, medium, and maximum levels of spontaneous 117V PrP deposition in aged uninoculated 117VV Tg 30 mice. Fixed brain samples from the cohort of aged uninoculated 117VV Tg 30 mice shown in Fig 1C and S1 Data were stained for abnormal 117V PrP deposition using anti-PrP monoclonal antibody ICSM 35. (Upper row) Brain from a 117VV Tg30 mouse culled at 604 days old showing minimum (Min) pathology with sparse 117V PrP deposition in the anterior commissure, minimal deposition in the hippocampus, and no deposits in cortex and thalamus. (Centre row) Brain from a 117VV Tg30 mouse culled at 762 days old showing medium (Med) pathology with more substantial 117V PrP deposition in the anterior commissure, hippocampus, and the appearance of 117V PrP deposits in the cortex and thalamus. (Bottom row) Brain from a 117VV Tg30 mouse culled at 852 days old showing the maximum (Max) levels of spontaneous 117V PrP deposition seen in the aged cohort of mice. All areas shown have substantial deposits of 117V PrP. Scale bar: 3.6 mm in the overview (A, F, K), and 130 μm in all high magnification images. ICSM, Imperial College School of Medicine; PrP, prion protein
(TIF)

S2 Fig Flow chart showing primary and secondary transmission of spontaneous 117V prion isolates from 117VSpont-A and 117VSpont-B mouse brain in transgenic mice. (A) The original uninoculated 117VV Tg30 mouse designated 117V^Spont-A^ was culled with neurological disease at 476 days of age. (B) The original uninoculated 117VV Tg30 designated 117V^Spont-B^ was culled without neurological symptoms at 734 days of age. Both 117V^Spont-A^ and 117V^Spont-B^ mice had spontaneous PrP plaques in the anterior commissure of brain. In the transmission series reported, 1% (w/v) brain homogenate was used for all inoculations. Mice were observed for clinical signs of prion disease using criteria described in Materials and methods. Post mortem brain from inoculated mice was examined for evidence of abnormal PrP propagation by IB and/or IHC examination. PrP, prion protein; IB, immunoblotting; IHC, immunohistochemistry
(TIF)

S3 Fig Overview of histological findings in 117VV Tg30 mice challenged with spontaneous 117V prion isolates. The panels show schematic drawings reflecting the overall spatial distribution and intensity of the gliosis or PrP deposition within the experimental groups. They are not meant to indicate precise representations of individual brains. * Definition of values for neuronal loss: NL0 (none): No neuronal loss; NL+ (mild): Drop out of single neurones either focally or within the Ammon’s horn (AH), leaving the AH continuity intact; NL++ (moderate): Focal or regional drop out, interrupting the continuity of the AH and creating a small to medium gap (up to 1/3 of the length of the AH); NL+++ (severe): Neuronal drop out leaving gaps of more than 1/3 of the AH’s length. Ratios represent the proportion of samples with the corresponding neuronal loss score. Note that gliosis variability within experimental groups is considerable. PrP, prion protein
(TIF)

S4 Fig Neuropathological scoring criteria in 117VV Tg 30 mouse brain following secondary passage of spontaneous 117V isolates. Details of these transmissions are shown in Table 2. Fixed brain samples from inoculated 117VV Tg 30 mice were stained for abnormal 117V PrP deposition using anti-PrP monoclonal antibody ICSM 35, or with Harris HE staining for spongiosis, or GFAP immunostaining for astrogliosis. (Upper panel) Neuronal loss: (A) mild (score +) with single neuronal loss, indicated by the arrowheads. (B) moderate loss, (score ++), leaving short gaps, as indicated by the arrowheads; (C) severe loss (score +++). In this example, the entire neuronal ribbon (Ammon’s horn) is fully depleted of neurones. The arrowheads mark the beginning and end of the former Ammon’s horn. The thin ribbon of nuclei is formed by reactive astrocytes. (Centre panel) Example of spongiform degeneration: (D) overview, with boxes representing the areas shown in panel E (cortex) and panel F, (hippocampus). All sections stained with HE. (Bottom panel) representative images of brains with mild (G) and severe, extensive (H) astrogliosis. Immunostaining for GFAP. Scale bar corresponds to 400 μm in panels A, B, C; 3.2 mm in panel D; 100 μm in panel E, F, and 2.5 mm in panels G, H. GFAP, glial fibrillary acidic protein; HE, haematoxylin and eosin; ICSM, Imperial College School of Medicine; PrP, prion protein
(TIF)

S1 Data Raw data used to plot the graph in Fig 1C (Graphical representation of the incidence and severity of spontaneous PrP plaque deposition in brain of uninoculated 117VV Tg30 mice versus age). PrP, prion protein
(XLSX)

## Abbreviations

AAO: age at clinical onset
BSE: bovine spongiform encephalopathy
CJD: Creutzfeldt-Jakob disease
GSS: Gerstmann-Sträussler-Scheinker
IB: immunoblotting
ICSM: Imperial College School of Medicine
IHC: immunohistochemistry
IPD: inherited prion disease
NaPTA: sodium phosphotungstic acid
NPC: National Prion Clinic
p.i.: post-inoculation
PK: proteinase K
PrP: prion protein
PRNP: human prion protein gene
