# Supplementary figures and images for: Spontaneous generation of prions and transmissible PrP amyloid in a humanised transgenic mouse model of A117V GSS
Source: PLoS Biol. 2020 Jun 9;18(6):e3000725. doi: 10.1371/journal.pbio.3000725 (PMC7282622; doi:10.1371/journal.pbio.3000725)

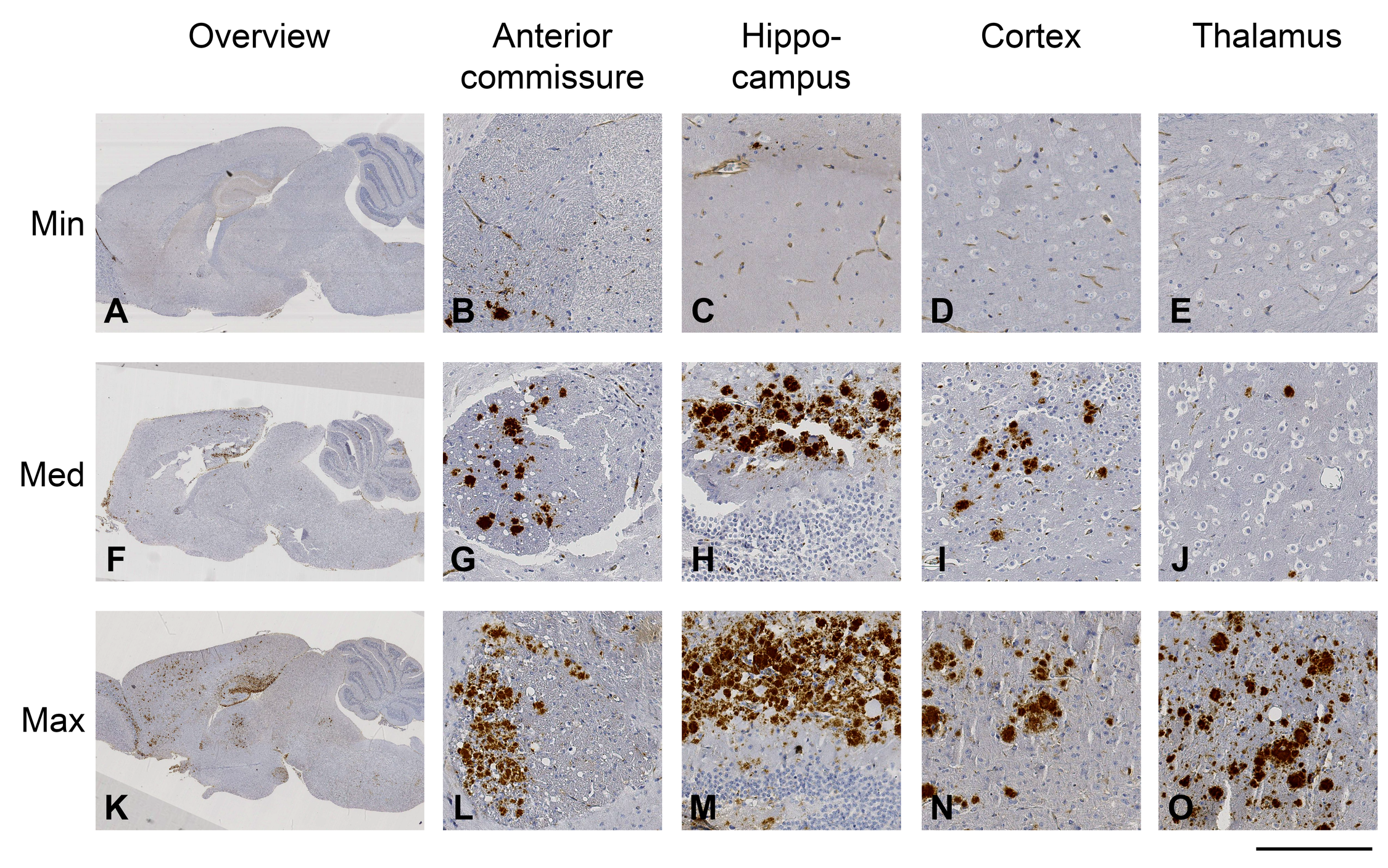

Supplement: S1 Fig — Fixed brain samples from the cohort of aged uninoculated 117VV Tg 30 mice shown in Fig 1C and S1 Data were stained for abnormal 117V PrP deposition using anti-PrP monoclonal antibody ICSM 35. (Upper row) Brain from a 117VV Tg30 mouse culled at 604 days old showing minimum (Min) pathology with sparse 117V PrP deposition in the anterior commissure, minimal deposition in the hippocampus, and no deposits in cortex and thalamus. (Centre row) Brain from a 117VV Tg30 mouse culled at 762 days old showing medium (Med) pathology with more substantial 117V PrP deposition in the anterior commissure, hippocampus, and the appearance of 117V PrP deposits in the cortex and thalamus. (Bottom row) Brain from a 117VV Tg30 mouse culled at 852 days old showing the maximum (Max) levels of spontaneous 117V PrP deposition seen in the aged cohort of mice. All areas shown have substantial deposits of 117V PrP. Scale bar: 3.6 mm in the overview (A, F, K), and 130 μm in all high magnification images. ICSM, Imperial College School of Medicine; PrP, prion protein (TIF) [file pbio.3000725.s001.tif]

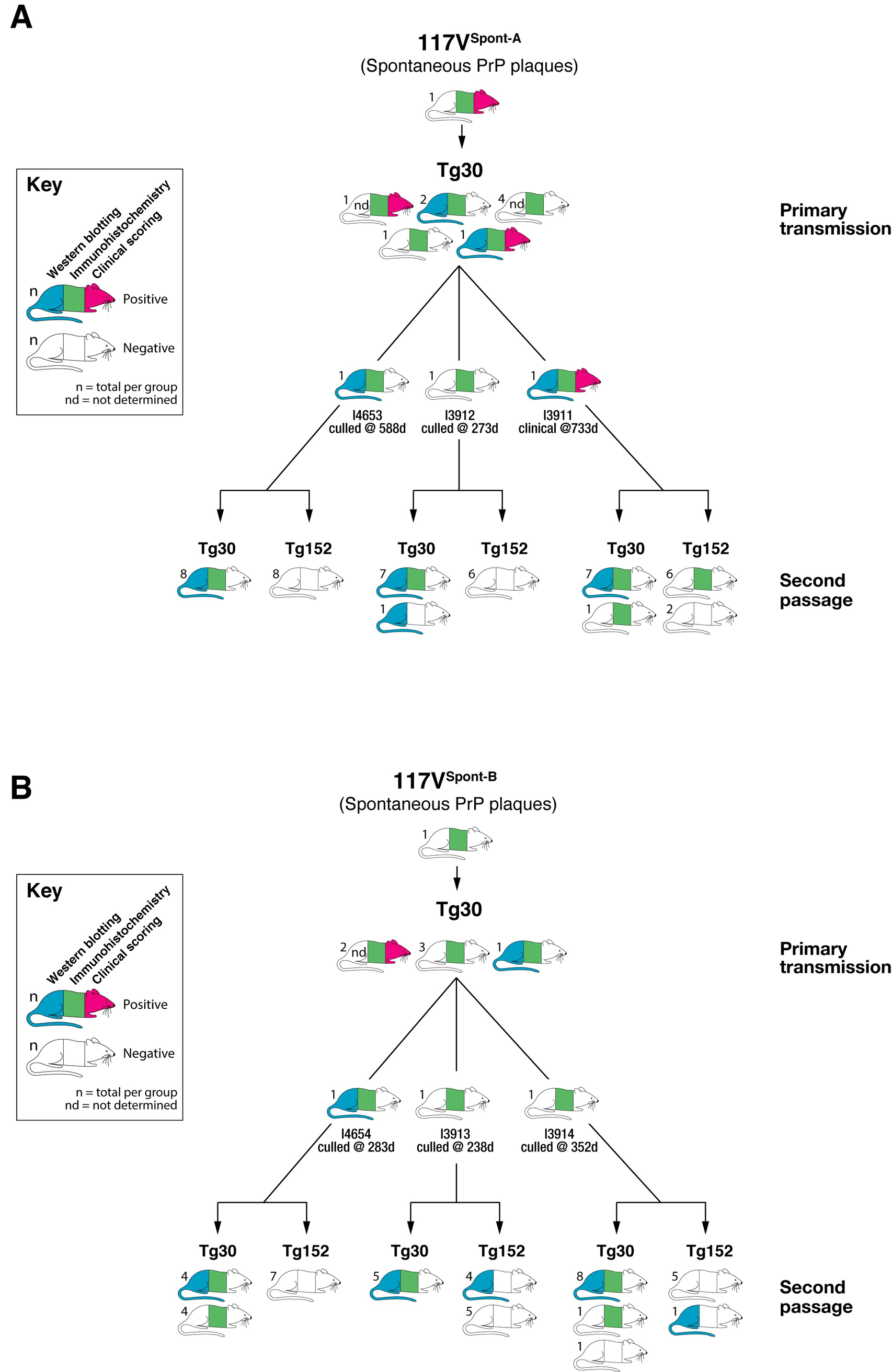

Supplement: S2 Fig — (A) The original uninoculated 117VV Tg30 mouse designated 117VSpont-A was culled with neurological disease at 476 days of age. (B) The original uninoculated 117VV Tg30 designated 117VSpont-B was culled without neurological symptoms at 734 days of age. Both 117VSpont-A and 117VSpont-B mice had spontaneous PrP plaques in the anterior commissure of brain. In the transmission series reported, 1% (w/v) brain homogenate was used for all inoculations. Mice were observed for clinical signs of prion disease using criteria described in Materials and methods. Post mortem brain from inoculated mice was examined for evidence of abnormal PrP propagation by IB and/or IHC examination. PrP, prion protein; IB, immunoblotting; IHC, immunohistochemistry (TIF) [file pbio.3000725.s002.tif]

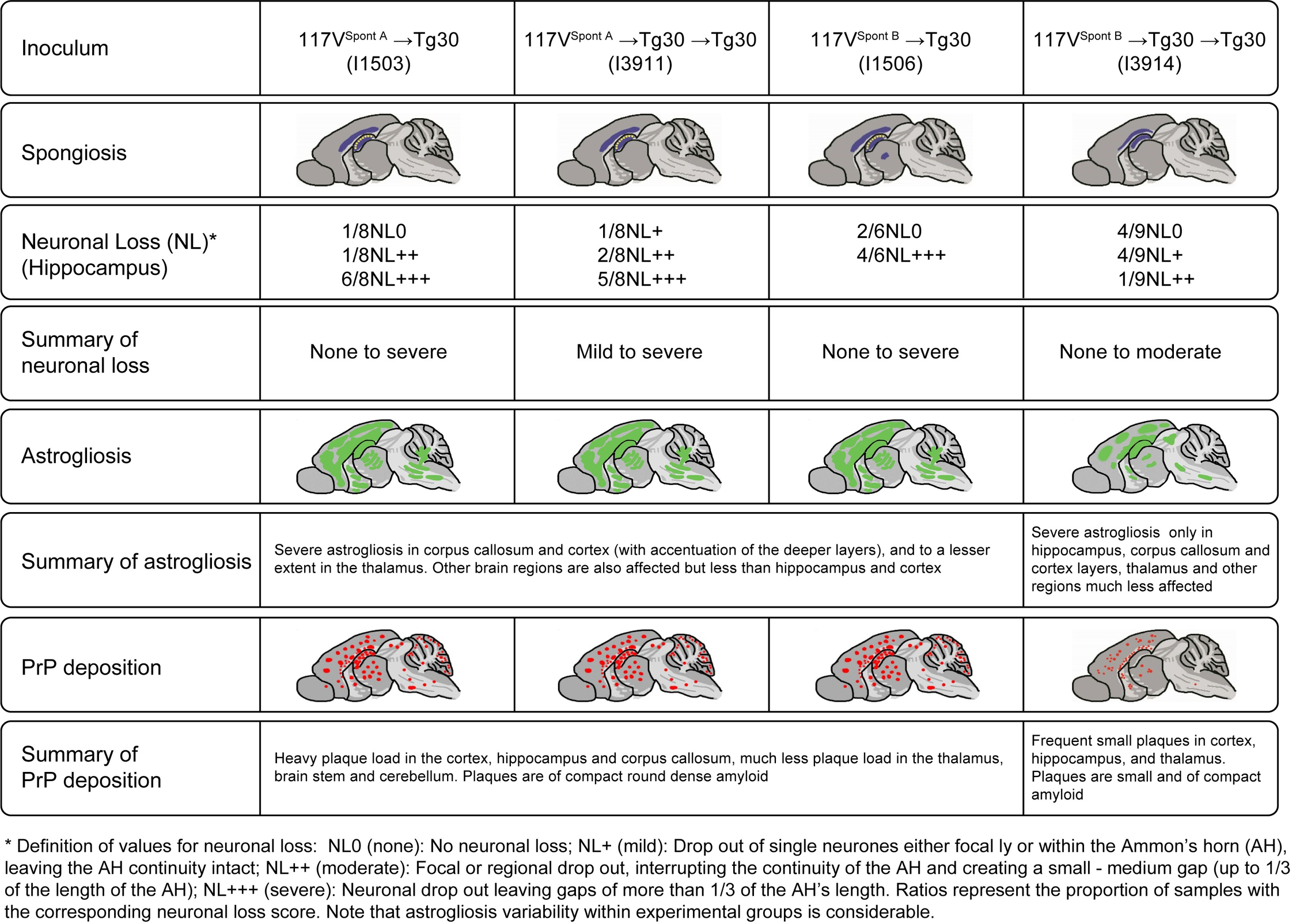

Supplement: S3 Fig — The panels show schematic drawings reflecting the overall spatial distribution and intensity of the gliosis or PrP deposition within the experimental groups. They are not meant to indicate precise representations of individual brains. * Definition of values for neuronal loss: NL0 (none): No neuronal loss; NL+ (mild): Drop out of single neurones either focally or within the Ammon’s horn (AH), leaving the AH continuity intact; NL++ (moderate): Focal or regional drop out, interrupting the continuity of the AH and creating a small to medium gap (up to 1/3 of the length of the AH); NL+++ (severe): Neuronal drop out leaving gaps of more than 1/3 of the AH’s length. Ratios represent the proportion of samples with the corresponding neuronal loss score. Note that gliosis variability within experimental groups is considerable. PrP, prion protein (TIF) [file pbio.3000725.s003.tif]

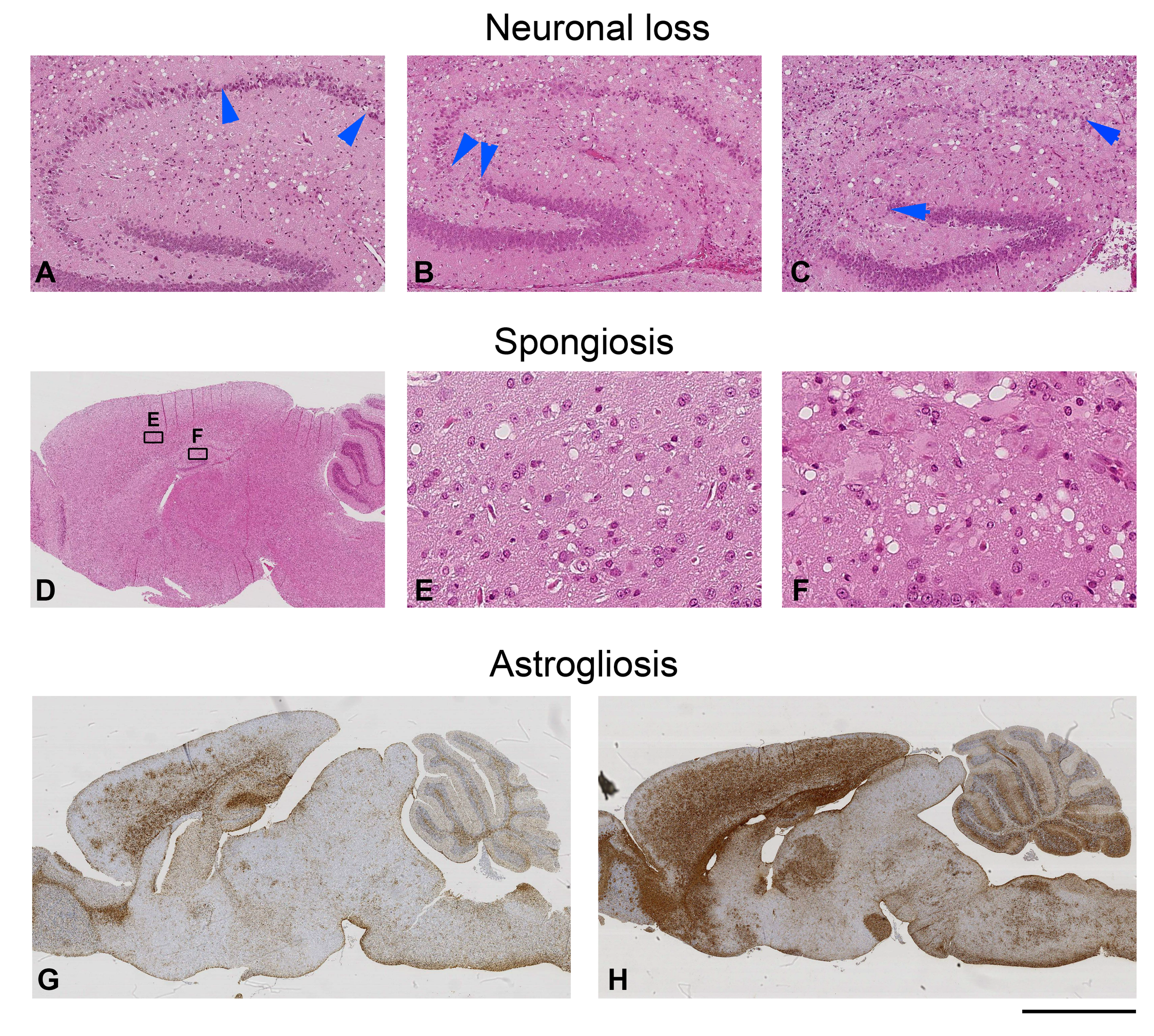

Supplement: S4 Fig — Details of these transmissions are shown in Table 2. Fixed brain samples from inoculated 117VV Tg 30 mice were stained for abnormal 117V PrP deposition using anti-PrP monoclonal antibody ICSM 35, or with Harris HE staining for spongiosis, or GFAP immunostaining for astrogliosis. (Upper panel) Neuronal loss: (A) mild (score +) with single neuronal loss, indicated by the arrowheads. (B) moderate loss, (score ++), leaving short gaps, as indicated by the arrowheads; (C) severe loss (score +++). In this example, the entire neuronal ribbon (Ammon’s horn) is fully depleted of neurones. The arrowheads mark the beginning and end of the former Ammon’s horn. The thin ribbon of nuclei is formed by reactive astrocytes. (Centre panel) Example of spongiform degeneration: (D) overview, with boxes representing the areas shown in panel E (cortex) and panel F, (hippocampus). All sections stained with HE. (Bottom panel) representative images of brains with mild (G) and severe, extensive (H) astrogliosis. Immunostaining for GFAP. Scale bar corresponds to 400 μm in panels A, B, C; 3.2 mm in panel D; 100 μm in panel E, F, and 2.5 mm in panels G, H. GFAP, glial fibrillary acidic protein; HE, haematoxylin and eosin; ICSM, Imperial College School of Medicine; PrP, prion protein (TIF) [file pbio.3000725.s004.tif]
